# Wireless fluorescence capsule for endoscopy using single photon-based detection

**DOI:** 10.1038/srep18591

**Published:** 2015-12-18

**Authors:** Mohammed A. Al-Rawhani, James Beeley, David R. S. Cumming

**Affiliations:** 1 School of Engineering, University of Glasgow, Oakfield Avenue, Glasgow G12 8LT, UK

## Abstract

Fluorescence Imaging (FI) is a powerful technique in biological science and clinical medicine. Current FI devices that are used either for *in-vivo* or *in-vitro* studies are expensive, bulky and consume substantial power, confining the technique to laboratories and hospital examination rooms. Here we present a miniaturised wireless fluorescence endoscope capsule with low power consumption that will pave the way for future FI systems and applications. With enhanced sensitivity compared to existing technology we have demonstrated that the capsule can be successfully used to image tissue autofluorescence and targeted fluorescence via fluorophore labelling of tissues. The capsule incorporates a state-of-the-art complementary metal oxide semiconductor single photon avalanche detector imaging array, miniaturised optical isolation, wireless technology and low power design. When in use the capsule consumes only 30.9 mW, and deploys very low-level 468 nm illumination. The device has the potential to replace highly power-hungry intrusive optical fibre based endoscopes and to extend the range of clinical examination below the duodenum. To demonstrate the performance of our capsule, we imaged fluorescence phantoms incorporating principal tissue fluorophores (flavins) and absorbers (haemoglobin). We also demonstrated the utility of marker identification by imaging a 20 μM fluorescein isothiocyanate (FITC) labelling solution on mammalian tissue.

White light endoscopy (WLE), has been a standard technique for diagnosis of disease pathology in the upper and lower part of the gastrointestinal (GI) tract for several decades^1^,^2^. However, until recently, the small bowel was an obscure region requiring invasive intervention for diagnosis and treatment. This changed after the approval of capsule endoscopy (CE) for medical use by the US Food and Drug Administration (FDA) in 2001^3^,^4^. Similar to WLE, CE uses white light imaging (WLI) and is potentially capable of viewing ailments including tumours, obscure gastrointestinal bleeding and Crohn’s disease within the small bowel^3^,^5^,^6^. However, both WLE and CE suffer from low detection rate. This drawback was overcome for the upper GI tract and duodenum by the introduction of multimodal imaging endoscopy that employs WLI, fluorescence imaging (FI) and narrow band imaging (NBI) in combination to significantly improve the detection rate from 53% to 90%^2^,^7^,^8^,^9^. New methods of improving detection rates within the lower part of the GI tract by means of software processing and 3D representation of captured WLI video are also being investigated. Robotic technologies to control capsule position and therefore enhance diagnostic and therapeutic capability are also being studied^10^,^11^,^12^. In this study, we focus on fluorescence imaging as a modality that has great promise for integration with current standard capsule endoscopy for the small bowel.

Fluorescence endoscopy exploits the natural phenomenon whereby specific molecules (fluorophores) absorb the excitation energy of blue light (380–500 nm wavelength) and then re-emit some of that energy in the form of green light (490–590 nm)^13^. These fluorophores can occur naturally within human tissue (endogenous) and are utilised in autofluorescence endoscopy, or can be introduced externally as labels to the biological system (exogenous) for use in targeted-fluorescence endoscopy^7^,^14^. Autofluorescence endoscopy (AFE) takes advantage of the fact that the concentration of endogenous fluorophores such as flavin adenine dinucleotide (FAD) and other extracellular matrices such as collagen and elastin in cancerous tissue can be up to three times lower than that of normal tissue^7^,^15^,^16^,^17^. An advantage of AFE is that it avoids introduction of foreign material, eliminating the risk of toxicity or other unwanted interaction with the biological system under investigation^14^. However naturally-occurring fluorophores occur in very low concentrations and exhibit very low quantum yield, limiting the effectiveness of AFE; for example FAD, the main contributor to autofluorescence emission exhibits a quantum yield of only 7%^18^,^19^.

An alternative approach, which enhances the effectiveness of fluorescence endoscopy, involves binding exogenous label fluorophores exhibiting very high quantum yield (e.g. 90% in the case of fluorescein isothiocyanate (FITC)) to areas of interest. By contrast to AFE, the fluorescent response from labelled diseased areas significantly exceeds that of surrounding healthy tissue, such that higher emission indicates a potentially diseased area, thus increasing the detection probability and specificity of early-stage abnormalities^13^,^14^,^20^,^21^. A fluorescently-labelled antigen which preferentially binds to tumours is introduced into a patient’s GI tract, thus making diseased areas more visible to a fluorescence-sensitive camera^14^,^22^,^23^,^24^. In the work done by^14^ a fluorescently-labelled peptide was developed which binds specifically to high grade dysplasia in the gut. Imaging of cancerous cells via binding of FITC to integrins on the cell surface has been demonstrated^25^,^26^. It has been shown that colon cancers can be imaged using two labelled mucins, one binding to cancerous cells, other to healthy cells^27^. The use of FITC-labelled dendrimers bound to cancerous cells in imaging has also been successfully validated^28^,^29^.

For investigations based on either endogenous or exogenous fluorophores, fluorescence endoscopes employ either a tungsten halogen light or short-arc xenon lamp source to generate the required narrowband (380–500 nm) excitation wavelength^17^,^23^,^30^ which is passed via an optical fibre bundle to the tip of the endoscope probe. Increasing illumination intensity will enhance imaging sensitivity, but will also tend to increase the rate of phototoxic reactions and photobleaching of the fluorophores themselves^19^. Hence FDA safety restrictions limit illumination power to 2 mW^14^. A very sensitive imager is therefore required to keep illumination power and fluorophore concentration low and within safe limits^14^,^23^.

Current fluorescence endoscopes use externally fitted charge-coupled device (CCD) imagers. These devices are not suitable for capsule integration since they are fabricated in specialised processes that preclude integration of the required interface electronics on to a single chip. CCDs are often cooled to increase signal to noise ratio and the corresponding sensitivity hence they are cumbersome and power hungry. Images are obtained via the aforementioned fibre-optic bundle probe that is capable of accessing only the oesophagus and duodenum. The optical system required for a FI system is also complex and difficult to implement within a capsule^31^,^32^,^33^.

Here we present a miniaturised wireless fluorescence endoscope that overcomes these challenges. Figure 1 shows in exploded form the capsule system that we have implemented and characterised. The device uses a range of technological innovations without which it would not be possible. We use a single photon avalanche detector (SPAD) array that is fully compatible with mainstream complementary metal oxide semiconductor (CMOS) technology. CMOS technology is an important method for miniaturisation and integration in biomedical applications^34^,^35^. A system-on-chip methodology has been adopted to deliver a chip that fully integrates the imager, high voltage power generation and the required addressing and data acquisition circuits. The highly sensitive SPAD generates a pulse in response to each photon impacting the active area of the device, thus allowing individual photons generated by autofluorescence to be counted. A cheap and compact light emitting diode (LED) is used for illumination at 468 nm – much simpler than in existing systems. The illumination power of the LED is only 78 μW, and the SPAD imager has sufficient sensitivity to work at these very low light levels. The device incorporates a miniature optical interference block that isolates the probe and fluorescence wavelengths, facilitating fluorescence imaging. The block contains optical collimation for the light source, the objective lens and filters. The system is completed with a wireless transmitter operating in the European industrial, scientific and medical (ISM) band at 868 MHz, and an external receiver pack. The final capsule relies on two 1.5 V button cells for power.

To illustrate a potential application of the integrated SPAD imager for capsule endoscopic fluorescence imaging we carried out two sets of experiments to validate the potential of using the capsule for autoflourscence imaging or targeted fluorescence imaging. Firstly, we imaged a phantom of endogenous fluorophores to demonstrate the potential for autofluorescence imaging (AFI) of tissue. Secondly we imaged exogenous fluorophores to illustrate the potential for targeted fluorescence imaging of areas of interest via fluorophore labelling.

## Results

### Optical system

An optical interference block is necessary to separate the excitation light source emission from the emitted fluorescence signal to be detected by the imager. Noise due to optical crosstalk must be minimised^13^. The block is designed to accommodate the excitation and emission wavelengths of both FAD fluorophore (460 nm excitation, 520 nm emission) and FITC (480 nm excitation, 520 nm emission) that are used in this work to prove the principles of autofluorescence and targeted-fluorescence imaging respectively. Other fluorophores have emission properties suitable for detection by a silicon SPAD, but FAD and FITC have the same emission wavelength and a similar excitation wavelength, so we could demonstrate the function of our device with the same choice of filters. A table of fluorophores and their optical characteristics is given in the supplementary section to illustrate our choice (see Supplementary Table 1). Clearly different embodiments of the device could choose different filter sets as required.

**Figure 2:**
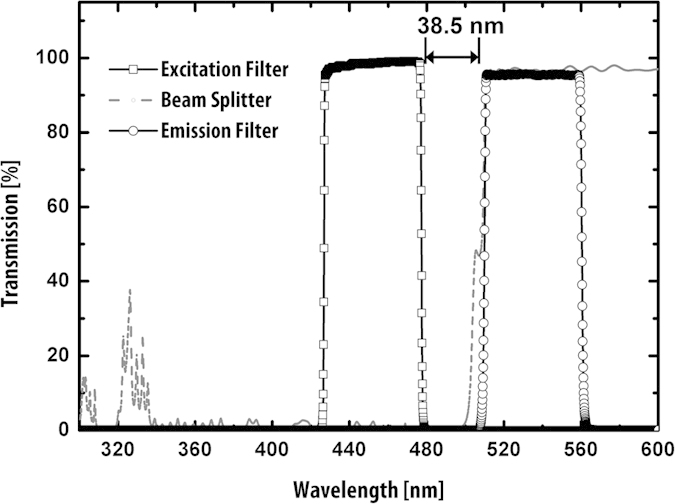
Optical block characteristics. Optical characteristics of filters and beam splitter.

The 11 mm H × 9.6 mm L × 8.6 mm W aluminium optical block incorporates an excitation source LED (468 nm, 15^o^ emission angle), a convex excitation lens with a focal length of f = 3 mm, a f = 10 mm achromatic objective lens, a 3mm diameter excitation filter (430–490 nm), a 9 mm diameter single-edge dichroic beam-splitter (506 nm), and a 9 mm fluorescence emission filter (513–555 nm). The spectral characteristics of the optical filters and beam splitter are shown in Fig. 2.

Supplementary Fig. 1. illustrates the optical block’s operation. Excitation light from the LED passes through the excitation filter that rejects the portion of the LED spectrum that extends into the fluorescence emission band. The filtered narrow LED beam is then focused by the excitation lens onto the beam splitter with an angle of incidence within the 45° ± 5° required for reflection. The reflected beam then passes through the objective lens resulting in a 67° illumination angle of the excitation beam at the object plane. The fluorescent emission from the sample is focused by the achromatic lens onto the SPAD imager’s active area. Crosstalk from the illumination wavelength is minimised by passing the fluorescent emission through the beamsplitter (which reflects the illumination wavelength) and fluorescence emission filter.

### Electronic system

**Figure 3:**
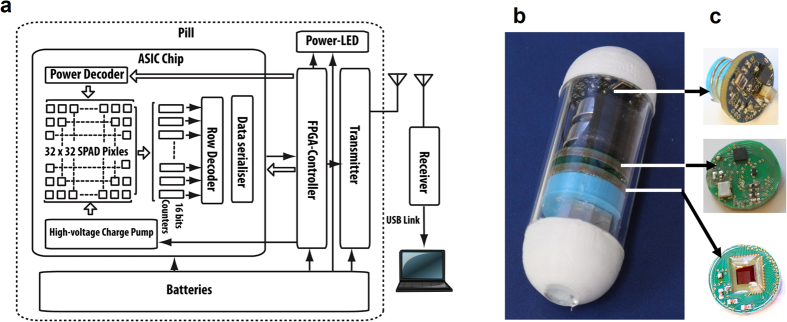
Electronic System. (**a**) Complete system block diagram illustrating the electronic configuration and radio link to the external base station. (**b**) Packaged pill. (**c**) 14 mm diameter PCBs hosting: (top) UHF transmitter and helical antenna, (middle) Lattice SiliconBlue FPGA controller and (bottom) imager ASIC.

The chip that forms the core component of the capsule is an application specific integrated circuit (ASIC) that incorporates a 32 × 32 single-photon avalanche diode (SPAD) array along with a charge-pump based power supply, addressing and pulse counting circuitry. The capsule also incorporates a low-power illumination LED, a Field-Programmable Gate Array (FPGA) controller and an ultra-high frequency (UHF) radio transmitter and antenna. The electronic system is illustrated in Fig. 3a. The operation of the capsule can be explained as follows: Illumination from the blue LED within the block causes the fluorophores within the tissue under examination to fluoresce. The resulting fluorescent emission is focused onto the SPAD imager’s active area, resulting in a series of pulses from each pixel that are counted by digital counters. The image obtained, consisting of an array of pulse counts, is read by the FPGA controller state machine and transmitted wirelessly to an external radio receiver, data logger and accompanying PC software that allow the images obtained from the capsule to be stored, displayed and processed (see supplementary Fig. 2 and note 1).

**Figure 4:**
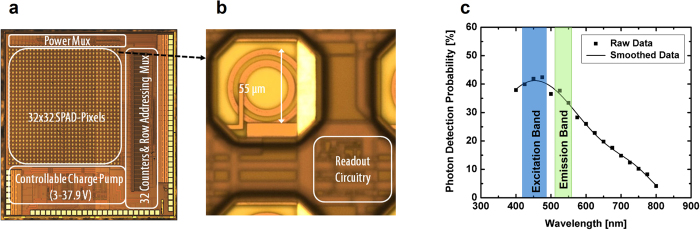
Capsule implementation. (**a**) Photograph of the 3.7 mm × 3.7 mm ASIC chip. The imager active area is 2.4 mm × 2.4 mm. The ASIC incorporates: 32 × 32 SPAD pixels, controllable charge pump (3–37.9 V), column multiplexer, 32× (16-bit) digital pulse counters and readout multiplexer. (**b**) Detail of a single SPAD pixel. (**c**) Measured PDP of the SPAD peaks at 475 nm which is suitable for autofluorescence emission between 490–590 nm.

The ASIC was fabricated in a 0.35 μm high voltage mixed-signal triple-well CMOS process (Fig. 4a). Each array pixel consists of an SPAD detector and readout circuitry (supplementary Fig. 3). The on-chip charge-pump generates the SPADs’ bias voltage, adjustable between 3–37.9 V by varying the frequency of an external digital clock, from the 3 V battery supply in the capsule^36^. Biasing the SPAD array above its 18.5 V breakdown voltage in this way causes the array to operate in Geiger mode whereby each SPAD generates a pulse in response to each photon arriving at its active area. The chip uses a “rolling shutter” readout scheme, in which one column of 32 SPADs is powered up at a time, and the resulting pulse output is counted by the 32 (16-bit) digital counters. A row decoder and multiplexer permit serial readout from the counters.

In order to demonstrate the technology’s suitability for use in capsule format, we packaged the optical, electronic and wireless systems and batteries into a 16 mm diameter, 48 mm length capsule as shown in Fig. 3b,c, and discussed in supplementary note 2.

### Electronic system performance evaluation

The ASIC’s performance was evaluated independently of the imaging system. In dark conditions, a single SPAD (Fig. 4b) has an average pulse count of approximately 12.3 kcps when biased by the charge pump at 4 V above its breakdown voltage for around 80% of the SPADs, while the remainder exhibit an average count of 45 kcps. Those pixels with a high dark count rate (DCR) are randomly distributed. The photon detection probability of the array (PDP: the probability of a photon impacting on a given SPAD’s active area resulting in an electrical pulse output) increases proportionally with increasing illumination intensity over a spectral bandwidth of 400 nm and 800 nm, as shown in Fig. 4c.^37^. At the fluorescence emission peaks of FAD and FITC (520 nm and 525 nm respectively) the SPAD shows a PDP in excess of 30% with no post process treatment of the silicon chip surface. When the SPAD array was illuminated directly using collimated white light it proved capable of detecting illumination levels as low as 19 pW/cm^2^ for an exposure time of 10 ms. The ASIC draws 1.79 mA on average at 3 V and 1 frames per second (FPS). The total power consumption of the capsule is 30.9 mW (see Supplementary note 3).

### Autofluorescence and fluorescence measurement

As an initial demonstration of our imager’s capabilities, we induced and detected fluorescence from a selection of readily available biological and manufactured materials. In addition to being present in human tissue, fluorophores also occur naturally in plant cells^38^. Office photocopier paper incorporates fluorescent brightening agents to improve whiteness^39^.

**Figure 5:**
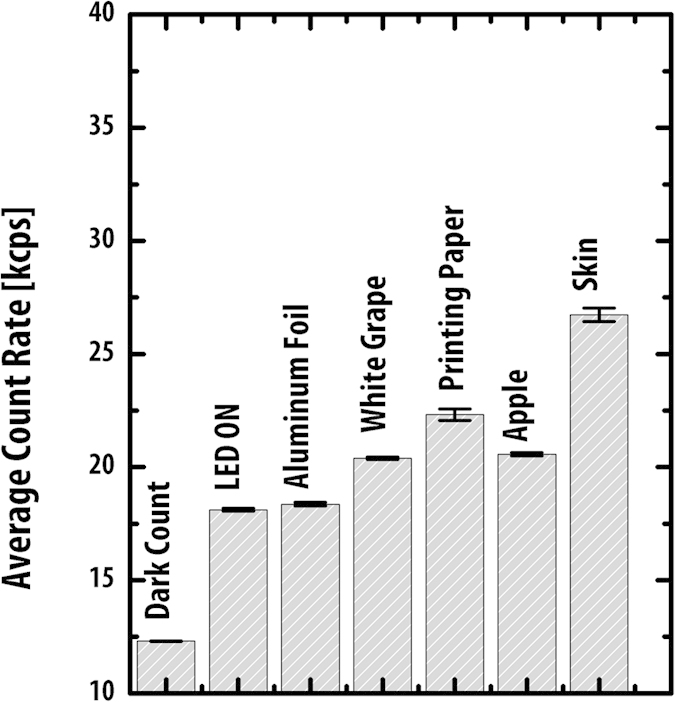
Fluorescence emission intensities of biological and manufactured materials.

The imager was placed in a dark environment at a distance of 11 mm from the sample under examination and the average count rate per second was recorded from each of the 1024 pixels. In order to determine the imager response due to optical crosstalk between the LED and detector, an initial measurement was taken with the illumination LED powered but no sample present, producing a photon count of c. 18.1 kcps. A sample of aluminium foil was then used to evaluate the imager’s response to reflected illumination light, resulting in a pulse count of c. 18.4 kcps. White grapes generated a pulse count of c. 20.4 kcps, an apple produced c. 20.6 kcps, while fluorescent emission from the skin of the thumb of one of the authors resulted in a c. 26.7 kcps photon count. White printer paper generated a c. 22.3 kcps response (Fig. 5).

Given the previously observed 12.3 kcps DCR measurement, the pulse count with no sample present indicates an optical crosstalk count of c. 5.8 kcps. The c. 0.3 kcps increase in response to the reflective foil sample over that obtained with no sample present indicates the imager’s response to reflected illumination light is modest. Given that the observed response from the foil is due to a combination of DCR, crosstalk and reflection, it is clear that any additional response in excess of this level from a particular sample is a result of induced fluorescence emission. This is indeed the case for the apple, grape, skin and paper samples, clearly demonstrating the imager’s capacity to induce and detect autofluorescence from both biological samples and a manufactured fluorescent brightening agent.

### Autofluorescence imaging with FAD

**Figure 6:**
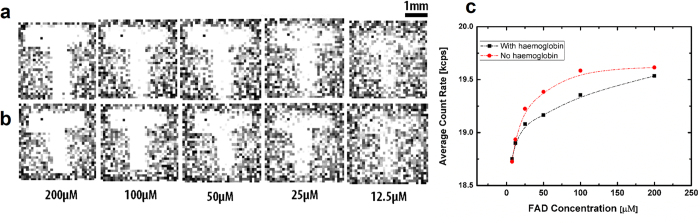
Results obtained using FAD phantoms. (**a**) Images of a feature (the letter T) taken by the capsule system for a 5 ml of FAD fluorophore solution at different concentrations, (**b**) shows images for same feature after adding 0.5 mg of haemoglobin to each one. Measurement at 10 ms SPAD gate time. (**c**) Average pulse count with and without haemoglobin absorption.

We demonstrate the autofluorescence imaging capability of our system by using FAD fluorophore to mimic the main endogenous contributor to the autofluorescence emission spectrum of human tissues^40^. 5 ml of a FAD solution was placed in a horizontally positioned polystyrene culture flask, the underside of which was covered in optically-absorbent tape with a 3 mm high T-shaped cutout (see supplementary Fig. 4a). The imager was directed upwards towards the cutout, which was positioned 22 mm from the capsule lens. With 78 μW excitation provided by the LED at the sample surface, 12.5 μM was the minimum concentration at which the T-shape was detectable (see supplementary Fig. 4a and Fig. 6a).

In AF detection, an excess amount of haemoglobin present within living tissue is considered an indicator of abnormality in the tissues as a consequence of neoplasia. As haemoglobin tends to be a photon absorber^41^,^42^, its presence reduces the amount of autofluorescence emission. To assess the impact of haemoglobin absorption, we added 0.5 mg to each (5 ml) FAD solution. The addition of the haemoglobin does not obviously alter the image at each concentration of FAD (Fig. 6b), but as can be seen in Fig. 6c, the average count rates of every image shows that haemoglobin absorption reduces the measured photon count.

### Imaging of exogenous fluorophores

As previously discussed, exogenous fluorophores may be attached to areas of interest in biological systems to enhance imaging capability.

**Figure 7:**
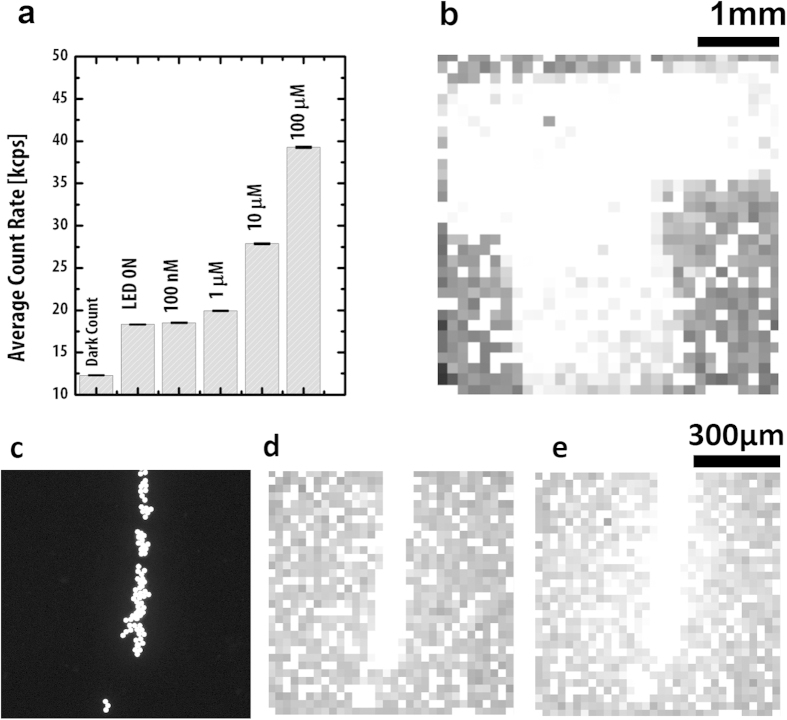
Results of fluorescence imaging on FITC solution and FITC coated beads. (**a**) A histogram of count rates for a 5 ml FITC at varying concentrations. (**b**) Image of a T-shape mask covering a sample porcine small-intestine immersed in 5 ml of a 20 μM FITC solution. (**c**) Image of fluorescent microparticles taken by fluorescence-enabled microscope. (**d**) Image of fluorescent microparticles taken by SPAD imager. (**e**) Image of microparticles placed on the surface of a sample of porcine intestine.

To assess the system’s capacity to image one such fluorophore, a 5 ml solution of FITC was measured at varying concentrations. The signal from a 100 nM FITC solution using a 10 ms gate time and 78 μW LED illumination is detectable (Fig. 7a). To demonstrate the ability to image a fluorescently labelled area of interest within a biological system, we used a sample of porcine intestinal tissue immersed in 5 ml of a 20 μM FITC solution, covered by a T-mask as described previously (Fig. 7b).

In order to model fluorescence imaging of fluorophores binding to areas of interest, we imaged FITC-coated 45 μm diameter polystyrene microparticles (Polysciences Europe, Germany). Figure 7c shows the image of a line of microparticles obtained from an Olympus BX51 microscope (Olympus Corporation, Japan) equipped with a cooled-CCD Hamamatsu C11440 camera (Hamamatsu, Japan). Figure 7d shows the image of the same line of fluorescent microparticles obtained from our imager. In order to demonstrate the ability to differentiate a fluorescently-labelled area from tissue, we injected a line of fluorescent microparticles on a glass slide, then placed a sample of porcine intestinal tissue on top (see supplementary Fig. 4b). The presence of the gut reduces the signal hence the phantom more accurately replicated the FITC concentrations that would be needed in practice. The SPAD array imager was directed upwards to obtain the image show in Fig. 7e.

## Discussion

We have demonstrated a highly miniaturised, low-power, wireless fluorescence imaging capsule with the potential for use in gastro-intestinal medicine. Lower power consumption is necessary for battery operation. We have successfully miniaturised a sensitive fluorescence imager, along with associated power supply and readout circuitry, on to a single CMOS chip, thus minimising size and component count. We have overcome the challenge of developing an imager with sufficiently low power consumption to be powered using small batteries by developing a photon-counting imager offering high sensitivity without the need for a power-hungry and bulky cooling system required by CCDs^43^. We have additionally developed low-powered wireless transmission, data acquisition and power management systems such that the entire system has an average power consumption of only 30.9 mW, and hence is capable of being powered by small batteries for sufficient time to traverse the human intestine.

Furthermore, the use of a CMOS SPAD has enabled our device to achieve sensitivity as low as 19 pW/cm^2^ using a 10 ms exposure time. This result compares favourably with a high performance CMOS photodiode with a sensitivity of 4 nW/cm^2^ 43. Owing to the sensitivity of the SPAD array, we are therefore able to use very low level illumination with a simple LED.

A major issue in fluorescence imaging, and a key aspect of high-sensitivity imaging, lies in separating the weak fluorescence emission from the illumination light. We have succeeded in miniaturising the optical filter system used in fluorescence microscopy to a size suitable for capsule use. As is the case for fluorescence microscopy, our experiments have shown that stray light from the illumination source is a contributor to the background level of our system. Although our miniaturised optical block implements band pass filters with a transmission of < 0.00008% at the filter stop band, some stray light that is not deflected by the beam splitter finds its way to the detector. The measured stray light count rate (SLCR) is c. 18.1 kcps (see Fig. 7a).

We have successfully demonstrated our system’s ability to induce and detect fluorescence from both exogenous and endogenous fluorophores above the 18.1 kcps SLCR floor. We have shown the ability to measure a solution of FITC fluorophore at concentrations as low as 100 nM in 5 ml samples. We have also demonstrated our system’s ability to image a fluorophore-labelled tissue sample by means of FITC-coated polystyrene microparticles placed on porcine intestinal tissue. We successfully imaged concentrations as low as 20 μM, a favourable sensitivity compared with the 100 μM concentration used in^14^. The experiment was conducted using the low illumination level emitted from the LED. As well as saving power, as discussed earlier, the low light level reduces the risk of phototoxic reactions and photo bleaching of the fluorophore^14^,^44^. The low illumination power (78 μW) yielded a signal-to-noise ratio (SNR) of 0.23 dBcr at 100 nM FITC concentration (the dBcr is defined to be 10 log_10_(CR/(SLCR), where CR is the count rate).

Our device has been extensively characterised against a range of materials, including human skin. In order to test the potential for using the device in the gut, we adopted the approach of using phantoms to validate the performance of the capsule in a similar manner to work described in^45^,^46^,^47^. We used FAD with a concentration similar to those described in earlier work^46^ and^47^. The results show that the minimum detectable concentration was 12.5 μM at an image-object distance of 2.2 cm using the 78 μW illumination intensity. Using these settings we found that the image sensor responded to increasing FAD concentration up to a maximum count rate of 19.6 kcps in a typical pixel. The signal saturation occurs when the available illumination can no longer excite further fluorescence (i.e. all the available light has been absorbed or scattered away). Whilst more light could be made available by increasing the illumination power, in practice the stray light in the system begins to dominate. The settings we use therefore represent an effective compromise between safe illumination levels, system power consumption, SNR, and wide molecular concentration range of sensitivity to FAD. We have also shown that presence of haemoglobin and the consequent optical absorption can be detected by our imager at concentrations as low as 0.01 g/ml. This result is comparable with the 0.012 g/ml minimum required to differentiate healthy and cancerous tissue as reported by^42^.

Development from a laboratory prototype into a commercially viable device would benefit from further performance enhancements in sensitivity, resolution and miniaturisation. There is potential to reduce the SPAD DCR with consequent improvement in SNR. Work presented by^48^ reported SPAD structures with a DCR as low as 100 cps. SNR may be further enhanced by introducing a light-absorbing “noise terminator” into the optical block to attenuate stray light from the illumination source^49^. Furthermore, sensitivity may be enhanced by introducing micro-lenses to the SPAD array, already common practice in CMOS-based imagers. It has been suggested that introducing micro-lenses can increase SPAD array gain by excess of a factor of 10^50^. The working distance of the imager from the sample is determined by the optical design. Future work would lead to optimisation of the optical geometry. Our data suggests that new labelling protocols using FITC to provide a positive tone contrast as opposed to a weaker negative tone contrast from FAD may be superior.

The imager’s 32 × 32 resolution is sufficient to demonstrate this new diagnostic tool, but is relatively low. The resolution may be enhanced by utilising a smaller feature-size ASIC technology.

Capsule size may be reduced by integrating the imager, controller and transmitter, presently implemented in separate chips onto a single ASIC, thus permitting the electronics to be integrated into a single PCB, as opposed to the 3 PCBs currently required. The optical block may be further miniaturised via use of more advanced computer numerical control (CNC) machining, spark erosion or laser sintering manufacturing technology. An antenna integrated into the capsule wall^51^, as opposed to the current PCB-mounted helix would also substantially reduce capsule size.

## Methods

### Optical block

The optical filter was designed using Solidworks and CNC-machined in aluminium to incorporate an LED (ASMT-BB20, USA, Avago Technologies), a convex lens (45117, Edmund Optics), achromatic lens (AC060-010-A, Thorlabs), an excitation filter (FF01-452/45, Semrock, USA), single-edge dichroic beam-splitter (FF506, Semrock, USA), and a fluorescence emission filter (FF01-534/42, Semrock, USA).

### ASIC

The ASIC chip was designed, simulated and laid-out using Cadence Virtuoso (Cadence Corporation, USA) and was fabricated using the commercially available AMS H35 high voltage process by AMS (Austria Microsystems, Austria). The fabricated ASIC was evaluated and the SPADs minimum detection limit was determined using a white light source driving an integrating sphere to create a uniform illumination. The SPAD was used to measure the intensity and thus determine the minimum detectable intensity that was then measured by an optical power meter (1936-R, Newport, USA). The same process was used to determine the minimum detectable intensity for the photodiode fabricated using the same process in a separate chip.

### Control and data logging

The image readout/transmission control state machine and charge pump clock divider were coded in VHDL, synthesised using Lattice IceCube software (Lattice Corporation, USA), and simulated/verified via Aldec ActiveHDL (Aldec Corporation, USA). Capsule end caps and the helical UHF antenna former were designed using Solidworks and manufactured by an Ultimaker 2 3D printer (Ultimaker Corporation, Netherlands).

Transmitted images from the capsule were captured by a receiver module consisting of a Melexis 71120 UHF receiver and end-fed dipole antenna (Melexis Corporation, Belgium), and ARM mbed embedded processor (NXP, Netherlands) and transferred via USB link to a Windows PC. A purpose-written MATLAB application provided real-time imaging and data logging, a second MATALB app permitted video playback and analysis.

### Imaging

All experiments for fluorescence measurements and imaging were conducted by placing samples at distances between 11 mm and 22 mm from the imager by means of a micropositioner (see supplementary Fig. 4).

The flavin solution was prepared by using FAD fluorophore (F6625, Sigma Aldrich) with phosphate buffer solution that was prepared using phosphate buffer saline (P5368, Sigma Aldrich). Five samples of 5 ml solution with concentrations varying from 12.5 μM to 200 μM were prepared in 25 ml polystyrene culture flasks. Similarly, the FITC solutions were prepared by using FITC fluorophore (46424, Sigma Aldrich) with sodium carbonate-bicarbonate buffer (pH 9). The mask for the T-shape was cut from absorbent tape (T743-2.0, Thorlabs) and taped to the flask.

## Conclusion

We have successfully demonstrated a miniaturised autofluorescence imaging system, sufficiently compact to be integrated into a capsule small enough to image the entire human GI tract, and offering power consumption low enough to allow up to 14 hours imaging and data transmission; sufficient to traverse the GI tract via peristalsis. We have successfully demonstrated imaging of models of both tissue autofluorescence and fluorescent labelling of areas of interest. These fluorophores produce opposing contrast mechanisms (i.e. dark or bright regions of interest) demonstrating two possible diagnostic methods. The new device creates the possibility of a diagnostic tool that will augment the already significant advances in clinical diagnostics for the GI tract that have been made using white light capsule endoscopy. Two modes of deployment are presently envisaged: that of using autofluorescence to identify abnormal pathology associated with tumours in the gut; and the use of markers to label malignant tissues. We have also identified directions for future enquiry that will lead to improved imaging resolution and further enhanced sensitivity. In successfully demonstrating a miniaturised fluorescence imaging capsule, we open up the possibility of fluorescence imaging of the human intestine below the duodenum.

## Additional Information

**How to cite this article**: Al-Rawhani, M. A. *et al.* Wireless fluorescence capsule for endoscopy using single photon-based detection. *Sci. Rep.* **5**, 18591; doi: 10.1038/srep18591 (2015).

## Supplementary Material

Supplementary Information

## Figures and Tables

**Figure 1:**
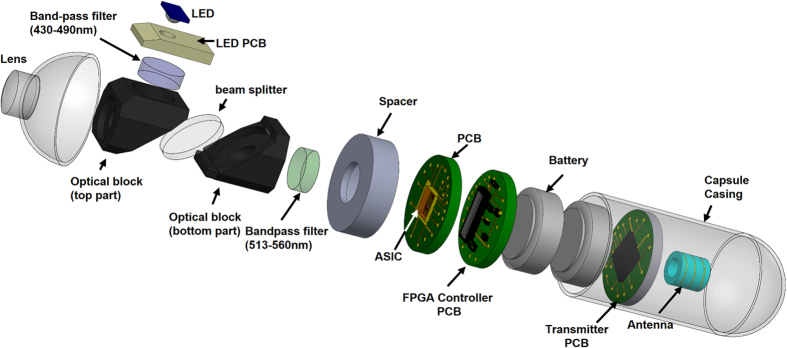
Exploded 3D view of the wireless fluorescence capsule endocope.
