# Supplementary material for: Wireless fluorescence capsule for endoscopy using single photon-based detection
Source: Sci Rep. 2015 Dec 18;5:18591. doi: 10.1038/srep18591 (PMC4683524; doi:10.1038/srep18591)
Supplement: Supplementary Information [file srep18591-s1.pdf]

Supplementary information

Wireless fluorescence capsule for endoscopy using single photon-based detection

Mohammed A. Al-Rawhani, James Beeley & David R. S. Cumming

School of Engineering, University of Glasgow, Oakfield Avenue, Glasgow G12 8LT, UK.

Supplementary figures

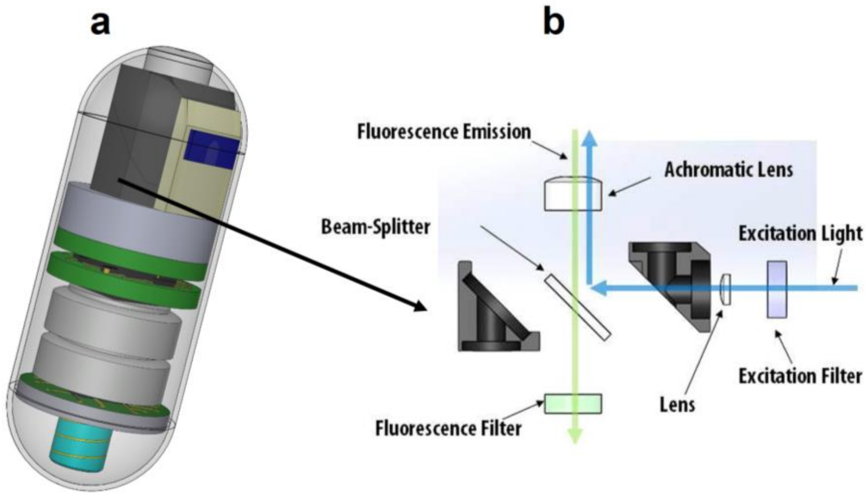

Supplementry Figure 1 | Optical block. (a) 3D CAD view of the capsule illustrating the optical block (b) Exploded CAD view showing optical block operation.

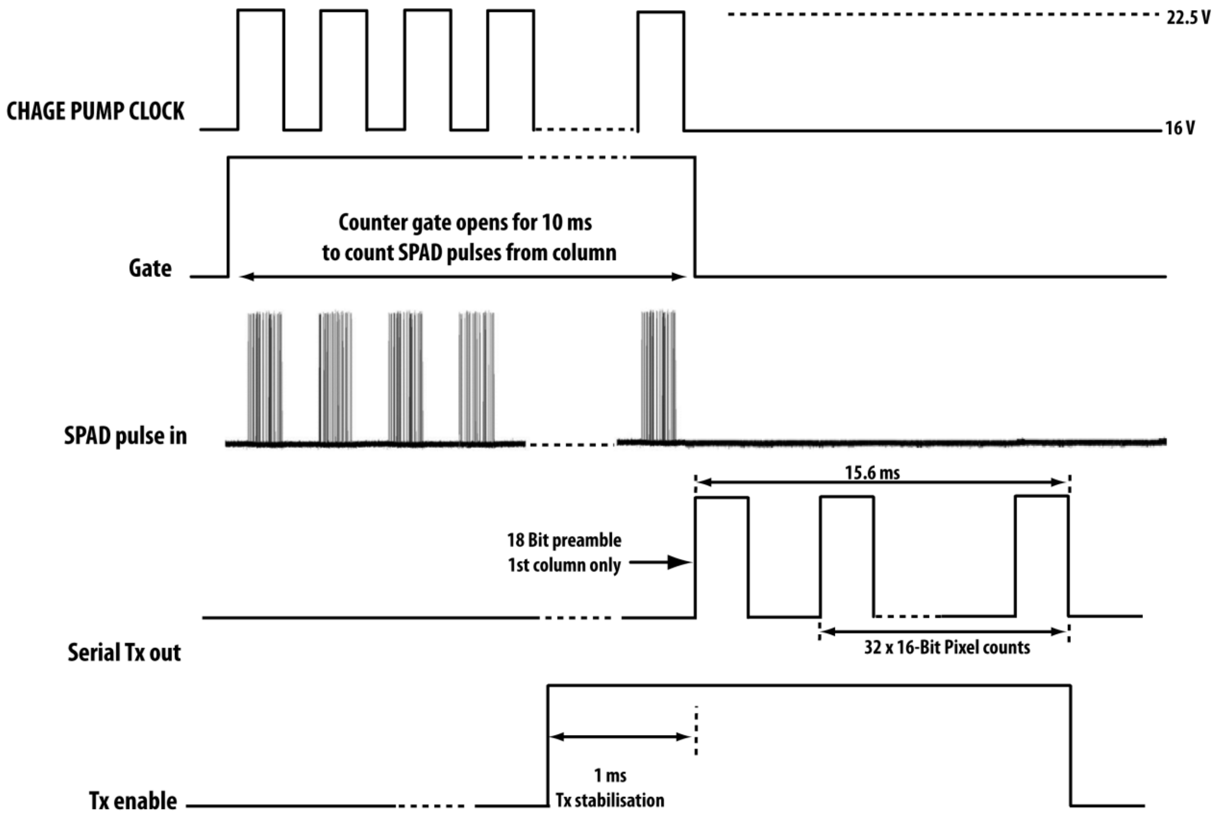

Supplementry Figure 2 | SPAD array readout and communication protocol. The 32 SPAD array columns are powered up and read out sequentially by the control FPGA state machine via the address decoder. The pulse output from each SPAD in the selected column drives one of (32) 16-bit digital pulse counters. The counter gate opens for 10 ms. After pulse counting the value of each counter is read serially by the FPGA. Data is transmitted asynchronously over a 32.768 kbit/sec, 868 MHz amplitude-shift keyed radio link. An 18-bit preamble prior to the first column indicates the start of a new image. The lowest 14 bits of each counter are transmitted, each preceded by 2 synchronisation bits required by the receiver for clock synchronisation. The transmitter is activated 1ms prior to transmission to allow it to stabilise. Transmission time for each array column is 15.6 ms, resulting in a total 819 ms readout and transmission time. One frame is acquired per second, acquisition being initiated by a 1 Hz clock from the FPGA.

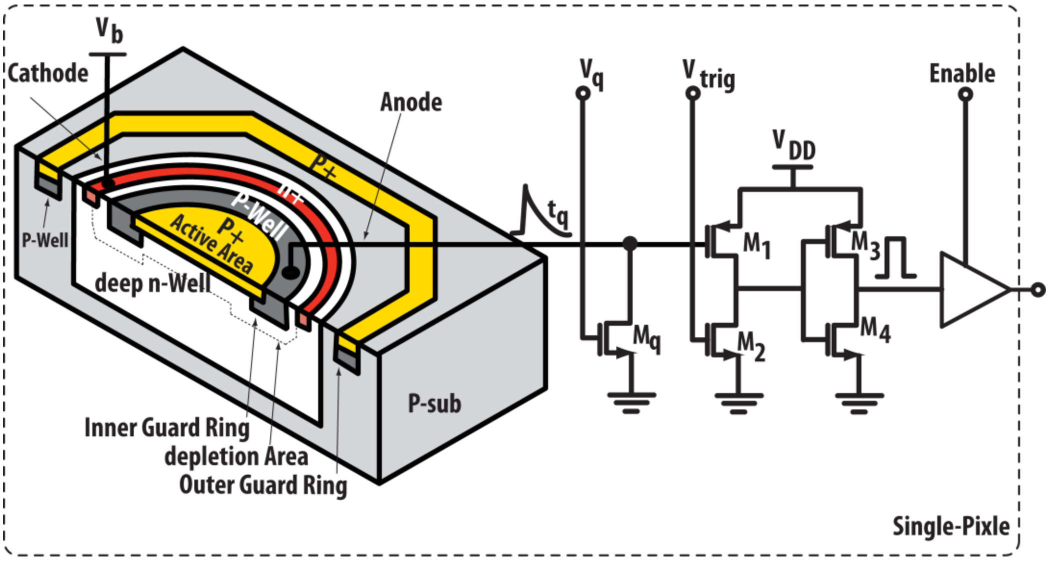

Supplementry Figure 3 | Single SPAD pixel. A cross section through the centre of a circular SPAD showing the guard ring of low doped material used to reduce the electric field at the edges and corners. The active area is 25 µm diameter, and the overall SPAD area including guard ring is 55 µm diameter. Each pixel incorporates a SPAD, an active quenching element M_q and controlled inverter (M_1 and M_2) followed by a buffer (M_3 and M_4) that drives a tri-state inverter.

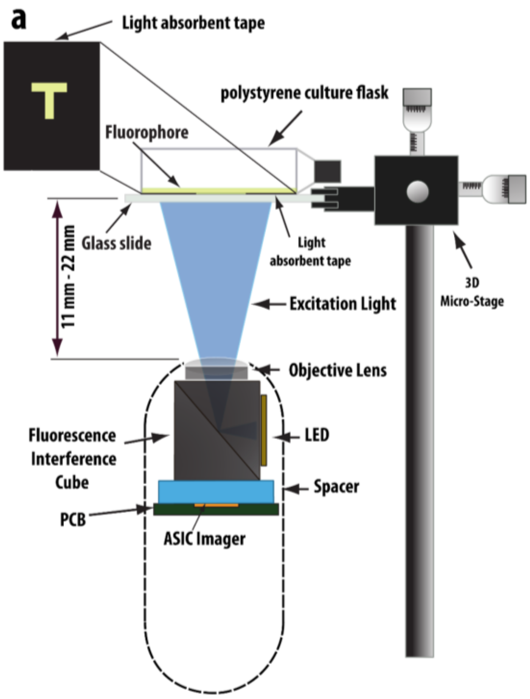

Supplementry Figure 4 | Apparatus for imaging fluorophore solution and labelled intestine sample. a) A polystyrene culture flask containing FTIC or FAD fluorophore solution is positioned above the upward-facing imager via a micro-stage. Optically-absorbent tape into which a T-shape is cut is attached to the underside of the flask as a mask. b) Porcine intestine placed over fluorescent microparticles laid on to a glass microscope slide is positioned above the imager via a micro-stage.

Supplementary Notes

Supplementary Note 1 - Radio frequency (RF) communication subsystem. The RF PCB implements a 1 mW 868 MHz amplitude-shift keyed (ASK) transmitter (Melexis 71120, Melexis, Belgium), and a 9 mm high, 3-turn normal-mode helical antenna on a 3D-printed former over a ground plane. The receiver/data logger located outside the body uses an end-fed half-wave dipole antenna. In order to validate antenna choice and data transmission reliability from the intestine to an external data logger a human body RF model consisting of a 48 cm diameter, 49 cm high plastic vessel containing a 0.9% saline (NaCl) solution was used [1]. By placing the capsule within the vessel and varying position and orientation relative to the receiving antenna, the communication subsystem was shown to be capable of reliable image transmission regardless of capsule orientation and consequent relative antenna polarisation.

Supplementary Note 2 - Design of wireless enabled capsule. The ASIC was bonded into an 0.8 mm deep milled recess in the 1.6 mm thick 14 mm diameter PCB FPGA (Fig. 3c (bottom)), with bond wires protected by an epoxy coating. A 1.6 mm high, 20-pin Molex Slimstack connector connecting to the controller PCB and off-chip charge pump capacitors are also implemented on the 6-layer PCB.

To control the ASIC and acquire the SPADs array count rates, we implemented a 14 mm diameter controller PCB incorporating a Lattice SiliconBlue FPGA (Fig. 3c (middle)). The data acquired by the FPGA is transmitted wirelessly to an external receiver and data logger PC by the transmitter and helical antenna are implemented on a further 14mm diameter PCB (Fig. 3c (top)).

The electronics, optical system and batteries were packaged in capsule formed from a borosilicate glass tube and 3D-printed polylactic acid (PLA) end caps. The final prototyped capsule weight including the two batteries is c. 14 g and is 16 mm in diameter and 48 mm in length.

Supplementary Note 3 - Power consumption. At 3 V, the LED consumes 5.2 mA. The ASIC PCB draws 1.79 mA, the FPGA PCB and transmitter PCB draws on average 2.93 mA. The complete capsule draws average power of only 30.9 mW. The capsule is capable of operating for 15 hours from the 3 V, 153 mAh battery back, in excess of the 12-14 hours typically required to traverse the intestinal system.

Supplementary Tables

Supplementary Table 1 - Excitation and emission wavelengths of some endogenous fluorophores in human tissues and exogenous fluorophore FITC [2-4].

Fluorophore	Optimal Excitation Wavelength (nm)	Peak Autofluorescence Emission (nm)
**Endogenous Fluorophores**		
Structural proteins		
Collagen	330	390
Elastin	360	410
Enzymes and coenzymes		
NADH	340	450
Flavins	460	520
NADPH	336	464
Vitamins		
Vitamin A	327	510
Vitamin K	335	480
Vitamin D	390	480
Vitamin B6 compounds		
Pyridoxine	332,340	400
Pyridoxamine	335	400
Pyridoxic acid	315	425
Prydoxal 5-phosphotae	315	425
Lipids		
Phospholipids	436	540
Lipofuscin	340-395	540,430-460
Porphyrins	400-450	635, 690
**Exogenous Fluorophore**		
FITC	480	520

Supplementary reference:

1. Godara, L. C. Handbook of Antennas in Wireless Communications (CRC Press, 2001).
2. Song, L. and Wilson B. Endoscopic detection of early upper GI cancers. *Best Pract. Res. Cl. Ga*. **19**, 833-856 (2005).
3. Ramanujam, N. Fluorescence Spectroscopy of Neoplastic and Non-Neoplastic Tissues. *Neoplasia* ***2***, 89-117 (2000).
4. Lichtman, J. W. and Conchello, J.A. Fluorescence microscopy. *Nat. Methods.* **2**, 910-919 (2005).
